# A Tool to Estimate the Risks of Repeat Cesarean Section

**DOI:** 10.1371/journal.pmed.0020325

**Published:** 2005-09-13

Cesarean section can be a life-saving technique for both mother and infant; however, it is a major abdominal operation that poses medical risks to a mother's health, including infections, hemorrhage, need for transfusion, injury to other organs, anesthetic complications, psychological complications, and a maternal mortality two to four times greater than that for a vaginal birth. The World Health Organization (WHO) has said that no country can justify having a cesarean rate greater than 10%–15%. Despite this advice, in the past 20 years, cesarean section rates have risen to nearly 25% in some countries.

To address rising rates of cesarean delivery, health authorities have encouraged women with a previous cesarean to attempt vaginal birth in subsequent pregnancies. However, studies have indicated an increased risk of serious adverse outcome among such women who attempt vaginal birth compared with a planned repeat cesarean delivery. This is due to a greatly increased risk of complications among women who attempt vaginal birth but ultimately are delivered by emergency cesarean section. Consequently, researchers have tried to identify women at low and high risk of failure for an attempted vaginal birth after a prior cesarean, but currently there is no validated antepartum tool to predict the risk of a failed attempt at vaginal birth among women with a prior cesarean delivery.

Now Gordon Smith and colleagues describe the development of a simple, validated model to predict the risk of emergency cesarean section among women attempting vaginal birth after a previous cesarean delivery. They also try to determine whether women at increased risk of cesarean were also at increased risk of uterine rupture, including catastrophic rupture leading to death of the infant.

The team studied 23,286 women with one prior cesarean delivery who attempted vaginal birth at or after 40 weeks of gestation. The population was randomly split into two groups, one on which the model was developed and the second on which it was validated. 

The researchers found that the following factors were associated with emergency cesarean section: increased maternal age, lower maternal height, male fetus, no previous vaginal birth, prostaglandin induction of labor, and birth at 41 weeks or 42 weeks gestation compared with 40 weeks. In the validation group, 36% of the women had a low predicted risk of caesarean section and 16.5% of women had a high predicted risk; 10.9% and 47.7% of these women, respectively, were actually delivered by caesarean.

The predicted risk of caesarean was also associated with the risk of uterine rupture in general, and of uterine rupture associated with perinatal death, and women who were at low risk of emergency cesarean section were also at low risk of uterine rupture, including catastrophic rupture leading to perinatal death—one of the principal concerns among women who have had a previous cesarean birth.

Despite the strengths of the present study, including the very large population size, studies using registry-based data have the weakness of inconsistent definitions, admitted the authors. Also the study lacked data on other risk factors for emergency cesarean delivery, such as body mass index, the indication for the previous cesarean section, and whether a previous vaginal birth preceded or followed the previous cesarean section.

Nonetheless, the findings offer a validated model for estimating the risk of emergency cesarean section among women with a prior cesarean delivery who attempt vaginal birth. The true worth of the model will become clear when other researchers test it.

